# Response to Jaeggi’s J.S. Torday, N.W. Blackstone and V.K. Rehan, a cell-centered alternative to mainstream evolutionary medicine? Review of ‘*Evidence-Based Evolutionary Medicine*’

**DOI:** 10.1093/emph/eoz027

**Published:** 2019-09-28

**Authors:** John S Torday, Neil W Blackstone, Virender K Rehan

**Affiliations:** 1 Department of Pediatrics, Harbor-UCLA Medical Center, Torrance, CA, USA; 2 Department of Biological Sciences, Northern Illinois University, DeKalb, IL, USA

*Evidence-Based Evolutionary Medicine* by Torday, Blackstone and Rehan [1] is based on contemporary medicine being evidence-based, whereas Neo-Darwinism provides no causal experimental evidence for vertebrate physiologic evolution. The principle criticism of the book is that it does not reference the contents of the book. We chose a non-referenced narrative style instead of referencing our numerous publications in support of cellular-molecular physiologic evolution that this book is based on. Suffice it to say that all of the statements in the book are founded on experimental evidence.

The framework of the modern synthesis often works remarkably well, but it is only predictive when all parameters (e.g. allele frequencies, population sizes and selective coefficients) are specified in advance. Can greater predictive insight into the evolutionary process be gained from other perspectives? A levels-of-selection framework may provide possibilities in this regard, while exploring the emergent properties of complex physiology may provide other possibilities. For instance, at its core, the modern synthesis relies on randomness for a phenotypic change to occur and be selected, while a molecular, cell and cell–cell communication-centered approach may allow greater prediction in this regard. *Evidence-Based Evolutionary Medicine* may thus provide a much needed ‘logic’ and mechanistic insight into evolutionary processes, including multilevel selection, evolvability and transgenerational inheritance. While perhaps in some regards, this approach is embryonic and contrarian to the Neo-Darwinian approach, broader perspectives are essential to the growing and vital field of evolutionary medicine.

**Conflict of interest**: None declared.
